# Empirical evidence of the impact of mobility on property crimes during the first two waves of the COVID-19 pandemic

**DOI:** 10.1057/s41599-022-01393-0

**Published:** 2022-10-14

**Authors:** Kandaswamy Paramasivan, Rahul Subburaj, Saish Jaiswal, Nandan Sudarsanam

**Affiliations:** 1 Directorate of Vigilance and Anti-corruption, Government of Tamil Nadu, Chennai, India; 2 Department of Civil Engineering, Indian Institute of Technology, Madras, India; 3 Department of Computer Science and Engineering, Indian Institute of Technology, Madras, India; 4 Department of Management Studies, Indian Institute of Technology, Madras, India

**Keywords:** Criminology, Sociology

## Abstract

This paper seeks to evaluate the impact of the removal of restrictions (partial and complete) imposed during COVID-19-induced lockdowns on property offences such as robbery, burglary, and theft during the milder wave one and the more severe wave two of the pandemic in 2020 and 2021, respectively. Using 10-year data of the daily counts of crimes, the authors adopt an auto-regressive neural networks method to make counterfactual predictions of crimes, representing a scenario without the pandemic-induced lockdowns. The difference between the actual and forecast is the causal impact of the lockdown in all phases. Further, the research uses Google Mobility Community Reports to measure mobility. The analysis has been done at two levels: first, for the state of Tamil Nadu, which has a sizeable rural landscape, and second for Chennai, the largest metropolitan city with an urban populace. During the pandemic-induced lockdown in wave one, there was a steep decline in the incidence of property offences. On removing restrictions, the cases soared above the counterfactual predicted counts. In wave two, despite the higher severity and fatality in the COVID-19 pandemic, a similar trend of fall and rise in property cases was observed. However, the drop in mobility was less substantial, and the increase in the magnitude of property offences was more significant in wave two than in wave one. The overall trend of fluctuations is related to mobility during various phases of restrictions in the pandemic. When most curbs were removed, there was a surge in robberies in Tamil Nadu and Chennai after adjusting for mobility. This trend highlights the effective increase in crime due to pandemic-related economic and social consequences. Further, the research enables law enforcement to strengthen preventive crime work in similar situations, when most curbs are removed after a pandemic or other unanticipated scenarios.

## Introduction

### Background

During the course of two years of the pandemic, multiple waves of the COVID-19 infection overwhelmed many countries and posed unprecedented challenges to governments. Policymakers and people involved in dealing with public health and safety issues at all levels had to constantly change their strategies and priorities, keeping pace with the dynamics of COVID-19 variants and infections. Government-induced stay-at-home (SAH) orders (or lockdowns) were globally adopted to contain the contagion. Depending on the severity of the pandemic, demographic divisions, local situational factors, and unique social settings, the lockdown mandates and their periods differed across various countries/regions with varying degrees of restrictions. These SAH regulations have drastically altered the lifestyles of billions of people worldwide. People confined themselves to their homes, leading to a sharp decline in vehicular and human mobility in public spaces. Besides the SAH orders, other restrictive orders, such as quarantining certain groups of people and constraining the movement of goods and services in containment zones, where there are higher infection levels, also markedly influenced public lives and the ontological and experiential sense of space and time.

In a span extending from 23 March 2020 to 31 December 2021, the state of Tamil Nadu (TN), India, battled the pandemic in two waves: the milder wave one in 2020 and the more severe wave two in 2021. Wave two was alarmingly intense, taking a much heavier toll on lives with a rapid increase of infections spreading to different corners of the state, both temporally and spatially. This two-year period provided an opportunity for a definitive empirical study of the government’s interventional mechanisms in containing the pandemic and its consequences on crime. The authors investigate the ramification of the SAH orders on mobility and how mobility influenced the recorded levels of property offences in TN in an environment where the victims of crime faced hurdles in lodging their complaints.

**Fig. 1:**
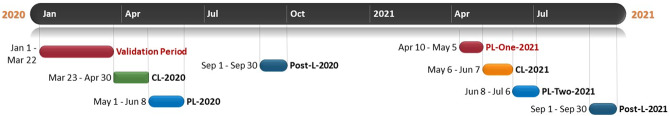
The timeline depicting different phases of lockdowns in wave one in 2020 and wave two in 2021 (CL complete lockdown, PL partial lockdown, Post-L post-lockdown).

Most studies have used descriptive statistical methods or conventional predictive techniques, while some have adopted deep learning forecasting tools to infer the causal impact of the pandemic-induced SAH orders on crimes. Notably, there are fewer articles based on primary data from peer-reviewed journals highlighting the pandemic’s effect on crimes in India. To address these gaps in the literature, the present research investigates the impact of the pandemic on property offences—the period covered two waves of the COVID-19 pandemic in multiple windows of varying degrees of restrictions in 2020 and 2021. This study relies on data obtained from official sources and an Auto-Regressive Recurrent Neural Network (ARNN) model to forecast the daily count of property offences in the various spans. This model outperforms other well-established forecast methods such as the Auto-Regressive Integrated Moving Average (ARIMA), Holt-Winters, Bayesian Structural Time Series Model, and the General Additive Model. The study also has two levels of analysis to check for the consistency of the trends and find any urban-rural differences between TN and Chennai, the capital and the largest urban city in TN. The research findings could help practitioners and policymakers appropriately prioritise resources during different phases of SAH orders, especially when restrictions are removed. The timeline of the study is detailed in Fig. 1.

### Literature survey and theory

Existing literature on the worldwide impact of SAH orders on property offences during the COVID-19 pandemic strongly indicates a global trend of the drastic reduction in property offences, albeit with uneven effects across types of property offences and geographical locations. For example, in a global study covering 23 countries across different continents, Nivette and others (2021) found that stringent restrictions on non-essential movement and activities were hugely influential in reducing burglary, robbery, theft, and auto-theft cases. Such decreases in property offences were observed across the spectrum in low, middle, and high-income countries, with variation in the magnitude and type of property offences. The studies also signal that, while property offences have declined, there has been a concurrent rise in violent offences such as cybercrimes and domestic violence, including child maltreatment and abuse.

Studies in high-income countries generally record a greater reduction in property offences as compared to violent crimes. For example, early studies of prominent cities in the United States (US) during the COVID-19 pandemic found a decline in residential burglaries during SAH orders, with one study observing an increasing trend of domestic violence (Mohler et al., 2020) and another noting no significant change in the case of assaults (Ashby, 2020). While a study by Scott and Gross (2021) recorded a steeper reduction in property offences as compared to interpersonal crimes in Baltimore, Chicago, and Baton Rouge during SAH-mandated periods. Another study of 29 prominent US cities found mixed trends, with no change in burglary and a sharp decline in robbery and larceny but escalations in auto theft and homicide (Meyer et al., 2022). A more recent study of four major US cities also indicated marked changes in acquisitive and violent crimes during COVID-19, including a decrease in property offences (Hou et al., 2022).

In England and Wales, crimes did not decrease uniformly during the first two years of the pandemic, with wide temporal and spatial variations observed. For example, property offences, especially shoplifting and robbery, decreased drastically, while public order offences and violent crimes escalated significantly (Agrawal et al., 2022). Halford and others (2020) studied the enforcement of SAH orders in a UK police force area using the mobility elasticity of crime, defined as a percentage change in crime due to a 1% change in mobility. They observed declines in several types of crime, including property offences. Notably, the second national lockdown imposed in England was less pronounced in reducing crime than the earlier lockdown (Farrell and Dixon 2021). Meanwhile, in Ireland, Buil-Gil and others (2021) observed that traditional offences like burglary, theft and violent and sexual crimes decreased during lockdowns but rebounded to pre-lockdown levels during post-lockdown (Post-L) periods. On the other hand, cybercrime in the Post-L period was higher than pre-COVID-19 levels (Buil-Gil et al., 2021).

Similar research findings were reported from middle-income countries. A study conducted among public transport passengers in Mexico City showed a clear link between the decline in seven categories of crimes and the drop in public mobility (Estévez-Soto, 2021). However, the authors noted that changes in the reporting behaviour of victims are often a determining factor in the decrease or increase in crime statistics, underscoring the need to study crime report trends (Estévez-Soto, 2021). In Rio De Janeiro, a study of COVID-19-induced SAH orders found that a decline in mobility strongly affected extortion and property offences but did not interfere with the dynamics of violent crime (Bullock et al., 2021). A similar study in Buenos Aires and Argentina found that an increased government presence in the form of health and social services caused a downward trend in acquisitive crimes (Perez‐Vincent et al., 2021). India also witnessed a sharp drop in visible crimes such as theft, robbery, and dacoity (Som et al., 2020). In contrast, in Nigeria (Akanmu et al., 2021), a spike in theft and rape during the COVID-19 lockdown was attributed to catalytic agents such as idleness, poor governance, and poverty.

Much of the existing literature studying the impact of pandemic-induced SAH orders employs routine activities theory (RAT) and crime opportunity theory to arrive at highly accurate explanations of altered crime trends. According to RAT, the occurrence of a crime event is a function of risk—that is, it is likely only when there is a simultaneously temporal and spatial convergence of the activities of an offender, a suitable target, and the absence of a capable guardian (Cohen and Felson, 1979). Crime opportunity theory suggests that an offender will make a rational choice to commit a crime if there is a high reward and low risk. COVID-19 pandemic-induced lockdowns imposed restrictions on freedom of movement, reduced human contact, and enforced social distancing and social isolation. This combination of factors is highly likely to introduce new negative stimuli, concomitantly removing the positive ones, thereby making people susceptible to crimes and delinquencies (Agnew, 1992).

Several COVID-19 pandemic studies demonstrate that RAT and crime opportunity theory are persuasive tools in interpreting the changes in crimes, particularly instrumental and property offences. For example, a study utilising machine learning techniques combined with the explanatory powers of RAT and general stress theory accounted for the changes in crime patterns in London and Sydney in 2020 (Li et al., 2021). In China, a paper studying the effect of pandemic-induced lockdown on eight types of acquisitive crimes in a medium-sized city revealed that crimes such as pickpocketing, residential burglary, and electro-mobile theft witnessed a higher reduction than theft from automobiles and automobile, motorcycle, and bicycle theft (Chen et al., 2021). The findings from this study, which use Google Mobility Community Reports, demonstrate that the differential impact on crime incidence is because of target distribution during the pandemic, corroborating the opportunity theory of crime. Another study on the implementation of SAH restrictions in New York City found a decrease in the incidence of residential burglary, grand larceny, and robbery in alignment with RAT (Koppel et al., 2022). Further, this research on property and violent crimes in the immediate aftermath of the pandemic in Los Angeles also corroborate the accuracy of RAT, as a study of containment policies in the city demonstrated that such policies had a more substantial impact on instrumental crimes than expressive crimes. This suggests that changes in instrumental and property crime trends are better predicted and explained by circumstances causing a reduction in social interactions. Comparatively, RAT is less effective in grasping the dynamics of expressive and violent crimes (Campedelli et al., 2020).

## Data and method

### Data

The research is based in the state of TN, India. It is the sixth most populous state and the eleventh most significant state in terms of area. Its size is comparable to Nicaragua or the state of Alabama in the US. The state has both rural and urban populations of around 78 million people. It is one of India’s wealthiest states and has the second largest impact on India’s gross domestic product (Reserve Bank of India, 2021).

In India, in 2019, 2,896,497 cases were registered in the pre-pandemic as against 168,116 cases in TN, which accounted for 5.8% of the total cases in the country (NCRB, 2020). These cases are punishable under the Indian Penal Code, which broadly includes offences against the human body, property crimes, and disturbances to public order. Offences such as crimes against women, children, the elderly, drugs, alcohol, and arms are separately dealt with under special and local laws. In TN, the more commonly reported crime categories are murder, attempted murder, rioting, aggravated assault, robbery, theft, burglary, cruelty by husband and relatives, rape, sexual harassment, and child sexual abuse. The daily frequency of these cases for two periods is indicated in Table 1. The first period covers the pre-pandemic (1 January 2010–22 March 2020) and pandemic periods (23 March 2020–31 December 2021).

**Table 1:** Descriptive statistics: central tendencies for all crimes in both pre-pandemic and pandemic periods.

Type of crime	Mean	Median	Mode	Std. dev.
*Pre-pandemic Period (1 January 2010 to 22 March 2020)*				
Bodily offences				
Attempted murder	8.824	8	9	4.201
Murder	5.019	5	4	2.620
Aggravated assault	81.779	79	76	24.865
Rioting	7.929	7	6	5.161
Unidentified dead bodies	12.075	12	13	5.858
Missing person	32.440	32	18	14.417
Property offences				
Murder for gain	0.392	0	0	0.710
Dacoity	0.472	0	0	0.793
Robbery	8.399	8	7	3.606
Burglary	14.686	14	15	5.682
Theft	54.185	55	59	15.893
Crime against women and children				
Child abuse	4.000	3	0	4.497
Rape	5.364	2	1	19.019
Sexual harassment	1.489	1	0	1.526
Domestic violence	4.859	4	3	3.448
Dowry related	0.323	0	0	0.658
*P andemic period (23 March 2020–31 December 2021)*				
Bodily offences				
Attempted murder	8.678	8	8	5.046
Murder	4.579	4	5	2.795
Aggravated assault	70.299	69	75	27.681
Rioting	5.482	4	4	4.351
Unidentified dead bodies	16.985	17	18	4.963
Missing person	54.891	57	63	20.885
Property offences				
Murder for gain	0.146	0	0	0.387
Dacoity	0.450	0	0	0.719
Robbery	8.334	8	9	4.440
Burglary	13.404	13	16	5.763
Theft	42.536	45	48	17.111
Crime against women and children				
Child abuse	12.903	12	16	6.121
Rape	1.268	1	1	1.192
Sexual harassment	1.961	2	1	1.611
Domestic violence	2.271	2	2	1.862
Dowry related	0.492	0	0	0.741

The pandemic and the pandemic-induced lockdowns had differential impacts on various crime categories. Crime cannot be over-generalised and each crime category is unique in its own way. In violent crimes such as murder, aggravated assault, rioting, attempted murder, physical crimes against women, and child sexual abuse, the victims sustain bodily injuries. They invariably immediately seek the assistance of the hospital and ambulance services for relief. During the pandemic, especially in wave two, health and other allied infrastructure were overwhelmed with COVID-19 patients. Medical professionals were overstretched and thoroughly exhausted from attending to the infected people. Victims also avoided seeking the assistance of these first responders for fear of contracting an infection. Further, the first responders, especially the police and fire and rescue services, had taken the lion’s share of the commitment and helped each other in addressing the concerns of the public during the pandemic. Therefore, in this pandemic environment, more than mobility, the recorded levels of these categories of violent crime were significantly influenced by situational factors such as the ability, accessibility, and willingness of the victims of crime to seek the assistance of relief centres and the responsiveness and proficiency of the health care agencies. Hence, the authors prefer to focus on the present hypothesis of the research to study the relationship between mobility and property offences during a pandemic, as registered cases of other categories discussed above were more determined by situational factors than mobility.

Though dacoity and murder for gain are property offences, they are omitted from this research as no case was reported on most days of the week (mean less than one and mode equals zero). Therefore, the research primarily focuses on the three significant offences, namely theft, burglary, and robbery.

When a victim makes complaints about these offences, a First Information Report (FIR) is lodged at the police station, which forms the first document to commence an investigation. With regard to online complaints and distress calls received, police take up an enquiry to redress the grievance of the complainant, but no formal investigation is carried out. Unlike in high-income nations, most properties are not insured in India. Hence, primacy is given to these cases for the detection and recovery of the stolen properties of the victims from the offenders. Further, the police is the primary agency empowered by law to take action on the complaints of property offences. The study relies on FIRs; FIRs registered daily in the 1356 police stations across the state are treated as time series univariate data. Similarly, for Chennai City (Chennai), data is collected from 115 police stations. The state of TN has a fair share of the rural population, whereas Chennai, the largest metropolitan city, is predominantly urban.

Thus, for the time periods mentioned in Fig. 1, the analysis is done for three property offences. Further, these time-series data may not be dependent on each other. Some correlation may exist on account of the overall criminal activity.

Generally, an algorithm built using machine learning and deep learning techniques is based on data. Typically, three data sets are associated with the model development: training, validation, and test (actual forecast) data sets. Usually, the training data set is larger than the other two as model parameters are estimated during the training stage. The model evaluates the training data repeatedly to learn the data behaviour. Validation data provides the first test to check how well the model predicts the unseen data. Some modellers carry out the model parameter tuning at this stage to improve accuracy and avoid over-fitting. The last data set is where the actual prediction is made for the research.

The historical data commences on 1 January 2010. Without any intervention, the training period was from 1 January 2010 to 31 December 2019. The authors acknowledge that population growth directly impacts annual crime counts, which is captured as the trend component of the data during the training period from 1 January 2010 to 31 December 2019, which is suitably reflected in the estimation of the predicted daily count of crimes during the pandemic period. The parameters of the model are estimated in the training period. Using the estimated parameters, the model is tested on unseen data from 1 January 2020 to 22 March 2020, which is known as the validation period. There is no tuning of the model parameters during the validation period. The period from 23 March 2020 to 31 December 2021 is the prediction period.

For investigation and analysis, the government-enforced SAH orders during both the pandemic waves in 2020 and 2021 fall into two categories. The most severe form is complete lockdown (CL), and the milder one is partial lockdown (PL). There was a complete cessation of all activities in the CL phase, where the movement of all types of vehicles and people was banned. All institutions, marketplaces, business houses, and shops, including retail outlets selling alcohol and other establishments, were closed entirely. People were asked to remain inside their homes until the lockdown restrictions were lifted. People were allowed to buy groceries and vegetables only during a limited window of a couple of hours. Social gatherings, including marriages and funerals, were severely restricted. There was an exception to the plying of vehicles dealing with essential goods and people involved in the pandemic work, especially relating to departments of health, police, and local administration.

During PL periods, there were gradual relaxations of the severe restrictions, which included opening specific key and core industries and establishments with skeleton strength of human resources and vehicles were allowed to operate during specified hours of the day. Shops dealing with essential goods were allowed to open for a longer time than during CL. Sale outlets dealing with alcohol were permitted for a limited opening period during the day. Social gatherings were allowed with a ceiling of 50 people. However, places of large congregations, such as stadiums, theatres, malls, and large markets, remained closed. Importantly, schools and colleges remained shut. Private taxis and public transportation such as coaches, buses, vans, and trains were not operational.

The lockdowns were differently enforced in the two pandemic waves in 2020 and 2021. In wave one, CL-2020 was introduced on 23 March 2020 and lasted till 30 April 2020. PL-2020 began on 1 May 2020 and ended on 8 June 2020. Both periods contained the same number of days. From 9 June 2020 to 31 August 2020, there were relaxations of restrictions in phases. Most curbs were removed on 1 September 2020, marking the beginning period of Post-L-2020, and it ends on 30 September 2020 as TN was freed from wave one of the pandemic in the following months.

During wave two of the pandemic in 2021, the government initially introduced a partial lockdown (PL-One-2021), which commenced on 10 April 2021 and lasted till 5 May 2021. This was immediately followed by a complete lockdown (CL-2021), which witnessed severe restrictions from 6 May 2021 to 7 June 2021. Some restrictions were subsequently relaxed, and the second PL (PL-Two-2021) was introduced from 8 June 2021 to 6 July 2021. Thus, during wave two, CL-2021 was sandwiched between PL-One-2021 and PL-Two-2021. The period beginning 7 July 2021 witnessed a series of gradual relaxations and finally paved the way for almost all curbs removed in the post-lockdown period (Post-L-2021) from 1 September 2021 to 30 September 2021.

The immediate and direct impact of the restrictive SAH orders was the reduced mobility of people and vehicles. Mobility is measured using insights from Google Community Mobility Reports, which collates data from all its users, especially handheld devices and other data available in the public domain. The basic principle of community mobility reports is based on the individual user presence, and time spent at specific location categories is collated to indicate activity, which reflects the mobility. Those accessing Google applications with smartphones or handheld devices are allowed recording of “location history” (Sulyok and Walker, 2020). The ubiquitous smartphones reveal a user’s activity in terms of location and time spent. These twin sources of information form the basis of this mobility tracking tool. Researchers worldwide involved in work related to the pandemic have extensively used this resource for their studies. Data are categorised into six spatial domains: ‘retail and recreation’, ‘parks’, ‘groceries and pharmacies’, ‘workplaces’, ‘transit stations’, and ‘residential areas’ (Ritchie, 2020). The changes in mobility are captured only in relative terms and not absolute numbers.

The percentage change in mobility is based on the number of visitors at a particular spatial domain relative to the baseline period. It represents the relative change in percentages compared to baseline days and not the absolute number of visitors. Baseline days represent a typical value for that day of the week, given as a median value over 5 weeks from 3 January 2020 to 6 February 2020 (pre-pandemic period). Significantly, the daily mobility changes are compared to the corresponding baseline figure “day”. For instance, Monday is compared with the baseline period’s median value of five Mondays. The comparison with a particular day of the week is critical because of the different routines followed by people on weekdays and weekends.

There are some limitations to the application of this mobility index. First, it is only a relative term or percentage change from the baseline, not absolute numbers. Second, data is primarily based on those accessing Google applications with various handheld devices, and it is not reflective of the actual number of visitors to the region. For instance, visitors to the park may not bring in their handheld devices; thus, the actual number of visitors may be under-captured. Also, some users may opt-out of location sharing or not sign in to the Google application (Kishore et al., 2021). Third, the baseline period covers only 5 weeks in 2020 and is not the actual representation of the past observations of a longer duration. The five-week base period cannot factor in the seasonality of the movement; hence, while doing long-term analysis, the data needs to be used with caution. Fourth, the population may vary further away from the baseline period due to relocations and remote working options. Fifth, Google learns the areas/places/locations, and this may change as information about these subjects gets updated.

Victims’ non-reporting and under-reporting of crime is another limitation of the data. Several research studies testify to the widely acknowledged problem of non-reporting or under-reporting of property offences to the police. The most commonly held belief is that notifying a crime is more of a hassle than its worth. The dominant theory talks about the victims’ rational decisions based on a cost-benefit analysis (Goudriaan et al., 2006). Victims assess the risks, problems, and difficulties associated with reporting and compare them with the benefits they gain from reporting (Felson et al., 2002; Gottfredson and Gottfredson, 1988; Skogan, 1984). Other reasons attributed include a lack of faith in the police and viewing them as unfair, incompetent, and unsympathetic (Felson et al., 2002; Baumer and Lauritsen, 2010). Poor police–public relationship (Mbewu et al., 2021) is considered a prime reason for non-reporting offences. Fear of reprisal from the offender in a particular social environment is a significant hurdle to free reporting a crime (Singer, 1988). This limitation of the data does not pose a serious problem to the study as these factors were present before and during the pandemic as well as in post-pandemic periods, and they do not influence causal inference.

### Method

The domain of this paper is a counterfactual analysis using time series forecasting. In simple terms, a model predicts future values based on past observed values. The time-tested decade-old techniques of forecasting time series such as ARIMA and exponential smoothing have produced consistent, stable forecasts; however, when there are rapid changes besides multiple seasonality and moving holidays, these methods are not suitable.

The landscape of time series forecasting has changed from conventional statistical methods to machine learning techniques and now to deep learning methods. Especially in the last two years, many newer methods in deep learning have emerged and have been successfully applied in various domains. The present paper makes forecasts relating to property offences using a recurrent neural network (RNN) based model RNN models and their variants have been exhaustively used in speech recognition (Graves et al., 2013), language modelling (Mikolov et al., 2010), and in generating text and video recognition and description (Donahue et al., 2017). It has been adopted in supply chain management (Carbonneau et al., 2008) and finance applications (Sirignano and Cont, 2019; Mäkinen et al., 2019).

**Fig. 2:**
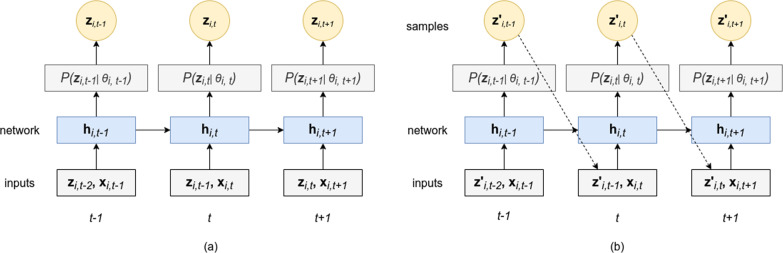
Architecture of DeepAR network. **a** The basic working mechanism of the DeepAR architecture. Here, *z*_*i,t−*1_ represents the input to the model and *x*_*i,t*_ represents an optional covariate at time step *t*. The input *z*_*i,t−*1_ is the target output at time step *t−*1. This study does not have any covariates. During training, the DeepAR model tries to learn the parameters by minimising the error between the output value and the target value, at the output layer. **b** Validation and Prediction. At each time step *t*, samples are drawn from the output distribution, and the median of the samples is passed as an input to the next time step *t* + 1. This process is continued till the prediction length is reached.

In this study, the authors use the GluonTS DeepAR model (Alexandrov et al., 2020), which uses an ARNN architecture to make forecasts for property offences data. The package implements the DeepAR model as proposed by Salinas et al. (2020). The model architecture consists of two parts as shown in Fig. 2a, an RNN-based architecture like long-short term memory (LSTM) or gated recurrent units (GRUs) to model the time-series data, and a likelihood function like Gaussian or negative binomial at the output to generate probabilistic forecasts. The advantage of having a probabilistic forecast is that it adds probabilities for different events and gives better reliability and confidence, giving DeepAR an edge over the existing statistical single time-series forecasting models.

The authors train the DeepAR model on three property offences data: burglary, robbery, and theft. For training the model, the authors use data corresponding to the property offences observed during 2010–2019 in the state of TN and in Chennai. The aim is to analyse the effect of pandemic-induced lockdowns on property offences during the two waves of the COVID-19 pandemic. The following describes the primary working mechanism of the model.

Let *i* represent the *i*th time series. At each time step *t*, the model receives an input *z*_*i,t−*1_ from the previous time step along with some optional covariates *x*_*i,t*_ at time *t*. This information gets propagated further to the RNN layers having parameters *ϕ*. The RNN layers receive this information along with the input from the hidden state *h*_*i*,*t*−1_ of the previous timestep and the output of the RNN layer *h*_*i,t*_, which is calculated as shown in Eq. (1), is used to estimate the parameters of the likelihood function.

1 $$h_{i,\,t} = h\left( {h_{i,\,t - 1},z _{i,t - 1},x_{i,t};\,\phi } \right)$$

As the property-offences data are counts, the negative binomial likelihood function, with a mean (*μ*) and the shape parameter (*α*), is used at the output. The parameters are calculated during the training process as shown in Eqs. (2) and (3), where $W_\mu ^{\rm {T}}$, *b*_*μ*_, $W_\alpha ^{\rm {T}}$, and *b*_*α*_ are the estimated parameters during the training process:

2 $$\mu \left( {h_{i,t}} \right) = {\rm {log}}\left( {1 + {\rm {exp}}\left( {W_\mu ^{\rm {T}}.h_{i,t} + b_\mu } \right)} \right.$$

3 $$\alpha \left( {h_{i,t}} \right) = {\rm {log}}\left( {1 + {\rm {exp}}\left( {W_\alpha ^{\rm {T}}.h_{i,t} + b_\alpha } \right)} \right.$$

The parameters of the network and the likelihood functions get updated during backpropagation. The training stops as the model parameters converge to optimal values.

After the DeepAR model parameters are estimated, forecasts are made for the given time-series data. At each time step *t*, samples are drawn from the output distribution, and the median of these samples is passed as input to the next time step, as shown in Fig. 2b; this continues till the prediction length is reached. This process is repeated *n* times, generating *n* different traces that are used to estimate the confidence intervals for the predictions made. The median prediction becomes more reliable as the value of *n* increases. The authors validate the forecasts made by the models on the validation data using the weighted mean absolute percentage error (WMAPE) metric as mentioned in Eq. (4). The advantage of using the WMAPE metric is that it handles the problem of getting an infinite error, like in mean absolute percentage error (MAPE) when the number of crimes is zero on a specific day. The predictions made by the model are then analysed for various stages of lockdown during waves one and two of the COVID-19 pandemic.

4 $${\rm {WMAPE}} = \frac{{\mathop {\sum}\nolimits_{i = 1}^n {\left| {{\rm {Actual}}_i - {\rm {Predicted}}_i} \right|} }}{{\mathop {\sum}\nolimits_{i = 1}^n {\left| {{\rm {Actual}}_i} \right|} }}$$

where Actual and Predicted refer to the actual and predicted value, respectively, at time step *i*.

The authors use the interrupted time series analysis technique to quantify the effect of lockdowns during the two waves of the pandemic. Cohen’s *d*, is generally used to calculate the effect size (ES) when the two distributions are normally distributed. The Shapiro test was used to test for the normality of the actual and the forecasted values. In case there was a violation of the normality assumption, instead of a *t*-test, the Wilcoxon signed-rank test was used to test the statistical significance of the differences observed in actual and predicted values. A non-parametric method called Cliff’s Delta was used to measure the ES (Hess and Kromrey, 2004). Owing to its non-parametric nature, Cliff’s Delta does not assume the nature of the distribution of the observations. To obtain the sample estimate for Cliff’s Delta, the actual and the predicted values are compared. The formula to calculate the sample statistic is given in Eq. (5), where *x*_*i*_ and *x*_*j*_ are the scores within the two groups—actual and predicted, and *m* and *n* are the sample sizes of the two groups, respectively. The symbol # represents the cardinality or the count.

5 $$\delta = \frac{{\# \left( {x_i > x_j} \right) - \# \left( {x_i < x_j} \right)}}{{mn}}$$

## Results

### Pandemic, Stay-at-home Orders and Mobility in wave one and wave two

This section deals with the impact of the SAH orders, enforced in multiple phases with varying degrees of moderation to curtail the spread of the virus during both waves in 2020 and 2021. Initially, the authors analysed how wave two of COVID-19 differed from wave one in 2020 in terms of severity, infection rate, transmission, and fatality rate. The restrictive measures were proportionate to the acuteness of the predicament. The mean/standard deviation/maximum number of infections per day during wave one and wave two were 2403/2223/6993 and 12,265/11,626/36,184, respectively; similarly, the fatalities per day were 34/36/127 and 153/163/483, respectively. The mean infection and fatality rates during wave two were nearly five to six times more severe than in wave one. During wave two, hospitals and other related health infrastructure faced a spate of infected people.

**Fig. 3:**
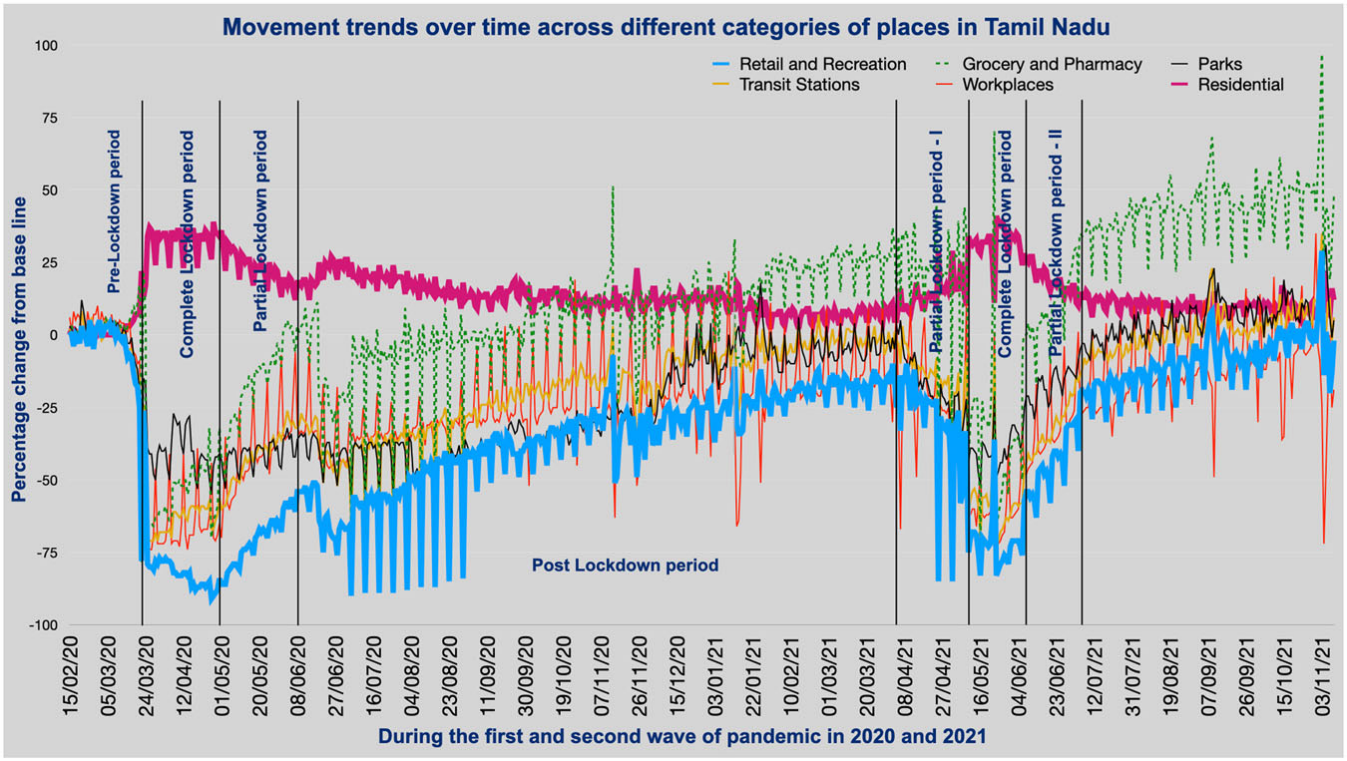
Percentage change in mobility from baseline in six community spaces (land use functions) is represented on the *Y*-axis, while the differing intensities of lockdown in two waves of COVID-19 are shown on the *X*-axis with date timeline (dd/mm/yy). The vertical bars indicate the phases of lockdown (CL complete lockdown, PL partial lockdown, Post-L is post-lockdown). Source: Community Mobility Report for Tamil Nadu, Google.

The restrictive orders of governments influenced the degree of mobility of people in varied activity zones; this impact had been in line with expectations. The authors have utilised the Google Community Mobility database for TN and Chennai for the relevant time period. There was a significant spike in the percentage change from the baseline value concerning residential neighbourhoods owing to the SAH orders. However, there was a steep decline in the percentage change in mobility from the baseline of all other spheres of activities, like retail and recreation, grocery and pharmacy, parks, transit stations, and workplaces (see Fig. 3). The quantum of this reduction was more substantial in CL and PL during wave one than in wave two. Retail and recreation, followed by workplaces and transit stations, witnessed the sharpest fall in mobility. The only sector to notice a hike was expectedly the residential area (see Table 2).

**Table 2:** Percentage change in mobility across six community spaces (land use functions) in Tamil Nadu and Chennai City during various phases of lockdown in both the pandemic waves in 2020–2021.

Place/period	Retail and recreation	Grocery and pharmacy	Parks	Transit stations	Workplaces	Residential
TN-CL-2020	−80.4	−52.21	−39.38	−62.03	−64.33	32.1
CNI-CL-2020	−88.87	−61.94	−88.48	−85.48	−78.6	36.38
TN-PL-2020	−70.21	−16	−40.82	−41.56	−39.21	22.77
CNI-PL-2020	−80.5	−39.3	−92.2	−75.5	−62.07	30.07
TN-POST-L-2020	−39.65	−0.57	−36.8	−22.7	−24.13	13.3
CNI-POST-L-2020	−51.7	−16.7	−68.7	−52.7	−45.9	17.01
TN-PL-ONE-2021	−47	−4.73	−34.04	−33.08	−36.19	19.42
CNI-PL-ONE-2021	−46.3	−1.69	−48.69	−40.42	−40.3	16.19
TN-CL-2021	−60.42	−22.15	−40.03	−47.67	−44.61	23.55
CNI-CL-2021	−79.3	−43	−76.18	−72.7	−66.8	31.03
TN-PL-TWO-2021	−35.07	9.31	−19.66	−23.97	−27.48	13.93
CNI-PL-TWO-2021	−54.82	−4.3	−55.9	−49.4	−49	19.37
TN-POST-L-2021	−6.8	45.6	9.16	4.2	−8.23	8.7
CNI-POST-L-2021	−23.83	24.23	−24.3	−16.6	−28	9.83

*TN* Tamil Nadu, *CNI* Chennai, *CL* complete lockdown, PL partial lockdown, Post-L post lockdown.

The authors study the factor of mobility in the six spatial domains of retail and recreation, workplaces, transit stations, grocery and pharmacy, parks, and residential as available in the Google Community Index in both TN and Chennai. There are two comparative studies of mobility. The first one compares two mobility distributions of CL-2020 in wave one and CL-2021 in wave two; there was a low to medium ES in terms of difference, where the reduction in mobility was more evident in wave one than in wave two. The second compares the two mobility distributions of the mild intervention period, PL-2020 in wave one and PL-Two-2021 in wave two. The difference, as expressed in large ES, maintains similar trends (see Table 3).

**Table 3:** Effect size for various group comparisons between various phases of lockdown in waves one and two in Tamil Nadu State and Chennai City.

Cliff’s delta effect size		PL-2020 vs. CL-2020	PLs-2021 vs. CL-2021	CL-2020 vs. CL-2021	PL-2020 vs. PLs-2021
Tamil Nadu	Retail and Recreation	0.548, Large	0.53, Large	−0.52, large	−0.73, large
	Grocery and Pharmacy	0.47, medium	0.51, Large	−0.35, medium	−0.85, large
	Parks	0.06, negligible	0.49, Large	−0.23, small	−0,84, large
	Transit stations	0.47, medium	0.47, large	−0.25, small	−0.35, medium
	Workplaces	0.56, large	0.54, large	−0.29, small	−0.35, medium
	Residential	−0.51, large	−0.5, large	0.14, small	0.44, medium
Chennai	Retail and recreation	0.53, large	0.527, Large	−0.59, large	−0.69, Large
	Parks	0.13, negligible	0.53, large	−0.67, large	−0.88, large
	Transit stations	0.49, large	0.50, large	−0.61, large	−0.76, large
	Workplaces	0.55, large	0.54, large	−0.43, large	−0.52, large
	Residential	−0.45, medium	−0.53, large	0.38, medium	0.65, large

*CL* complete lockdown, *PL* partial lockdown, *Post-L* refers to post-lockdown.

### Forecast accuracy of the model

From the results of the forecast accuracy of various models, the ARNN method has emerged as the most accurate model with the least number of errors (WMAPE) on validation data. It outperforms other forecasting models such as Holt-Winters, ARIMA, Bayesian Structural Time Series Model, and Generalised Additive Model in two out of three different time series relating to property offences (see Table 4).

**Table 4:** Validation error (WMAPE) for five models for the unseen data (1 January 2020–22 March 2020).

CRIME	ARIMA	GAM	BSTS	HOLT-WINTERS	ARNN
	WMAPE	WMAPE	WMAPE	WMAPE	WMAPE
Robbery	0.357	0.379	0.348	0.365	0.358
Burglary	0.316	0.470	0.319	0.449	0.288
Theft	0.274	0.364	0.278	0.337	0.141

*ARIMA* auto-regressive integrated moving average (ARIMA), *BSTS* Bayesian structural time series, *GAM* general additive model, *ARMM* auto-regressive neural networks, *WMAPE* weighted mean absolute percentage error.

### Crime trends in both waves of the pandemic—actual and predicted

All three property offences investigated show similar trends and patterns during the various intervention periods in both waves; however, there are variations in the magnitude of increase or decrease when compared with the counterfactual.

In TN, there was a substantial fall in robbery cases during CL-2020 and CL-2021, with a percentage change of −83% (ES −0.983) and −67% (ES −0.867), respectively. However, the decline was moderate during the PL-2020, PL-One-2021, and PL-Two-2021 with a change of −25% (ES −0.456), −6% (ES −0.21), and −14% (ES −0.30), respectively. When all curbs were removed, the recorded levels of robbery cases shot up by 30% (ES 0.389) in Post-L-2020 in wave one and considerably in wave two by 56% (ES 0.476). There was a similar pattern of a substantial fall during CLs, a moderate drop in PLs, and a sharp increase during Post-L phases in both waves for burglary and theft cases. There was a significant ES decline in CLs, a medium decrease in PLs, and a medium escalation in Post-Ls. Among the three offences, theft reported the highest magnitude of decline in all the phases (ES −1 in CLs, −0.988 in PL-2020, −0.76 in PL-One-2021, and −0.767 in PL-Two-2021). The maximum rise in actual cases when compared to counterfactual was noticed in robberies with 56% (ES 0.476) during Post-L-2021 (see Table 5).

**Table 5:** The percentage difference between actual and predicted registered property offences along with effect size (Cliff’s Delta) during various phases of lockdowns in both waves in 2020 and 2021 in Tamil Nadu.

Crime/Period	Robbery	Robbery	Burglary	Burglary	Theft	Theft
	% Change	Cliff’s delta (CI)	% Change	Cliff’s delta (CI)	% Change	Cliff’s delta (CI)
*Wave One*						
CL-2020	−83.10	−0.98 (−1,−0.88)	−68.91	−0.99 (−1,−0.96)	−80.17	−1 (−1,−1)
PL-2020	−24.98	−0.45 (−0.66,−0.20)	−46.62	−0.78 (−0.89,−0.56)	−53.66	−0.98 (−0.99,−0.96)
Post-L-2020	30.07	0.38 (0.11,0.54)	23.38	0.49 (0.23,0.69)	0.34	−0.09 (−0.28,0.13)
*Wave Two*						
PL-One-2021	−6.23	−0.21 (−0.54,0.14)	−18.93	−0.49 (−0.69,−0.13)	−23.84	−0.76 (−0.89,−0.41)
CL-2021	−67.03	−0.86 (−1,−0.46)	−58.73	−0.98 (−1,−0.92)	−64.47	−1 (−1,−1)
PL-Two-2021	−14.49	−0.30 (−0.55,0.00)	−26.97	−0.48 (−0.68,−0.22)	−35.08	−0.76 (−0.86,−0.60)
Post-L-2021	56.03	0.47 (0.18,0.67)	12.62	0.26 (0.02,0.5)	−10.19	−0.25 (−0.50,0.01)

*CL* complete lockdown, *PL* partial lockdown, *Post-L* post-lockdown.

**Fig. 4:**
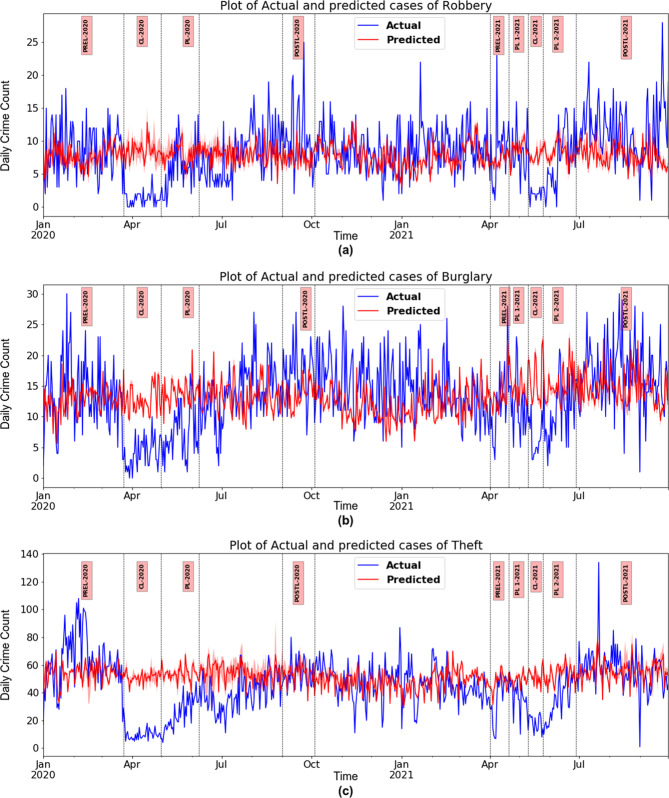
Impact of the first two waves COVID-19 on property offences. **a**–**c** Plot of the actual and predicted daily count of robbery, burglary and theft cases (on *Y*-axis) in Tamil Nadu during the differing intensities of lockdown in two waves of COVID-19 in 2020 and 2021 (on *X*-axis). The vertical bars indicate the phases of lockdown (CL complete lockdown, PL partial lockdown, Post-L post-lockdown). The prediction is done using auto-regressive recurrent neural networks.

Figure 4 shows a semblance of normalcy during the interim lull period between the two waves. During the last quarter of 2020 and the first quarter of 2021, the trend of the actual and predicted daily counts of theft and robbery cases was more or less the same. In contrast, the recorded levels of burglary were consistently higher than predicted in both waves, though moderately.

The analysis done for TN was replicated for Chennai, the state’s capital and largest metropolitan city. The declining trend in robbery cases in TN was noticed in Chennai in the same magnitude and direction during CL-2020 and CL-2021. The change in percentage of actual robbery cases when compared to counterfactual in Chennai during CL-2020 and CL-2021 were −75% (ES −1) and −78% (ES −0.879), respectively. However, during PL-2020 and PL-Two-2021, the reduction was more substantial in Chennai than in TN, with a declining percentage of −47% (ES −0.944) and −33% (ES −0.341), respectively.

The drop in burglary cases in Chennai during CL-2020 and CL-2021 followed the trend of TN with a slightly higher fall in the former period than in the latter as the percentage changes were −57% (ES −1) and −45% (ES −0.889), respectively. Similarly, during PL-2020 and PL-Two-2021, the corresponding declines were −37% (ES −0.889) and −13% (ES −0.22), respectively.

The decreasing trend of theft cases in Chennai was entirely synchronous in pattern with TN in all phases of both waves. The sharp fall of theft cases was 84% (ES -1) in CL-2020 and −66% (ES −1) in CL-2021; −61% (ES −1) in PL-2020; and −43% (ES −1) in PL-Two-2021. During the Post-L phase in wave two, escalation in robbery and theft cases were by 23% and 15.6%, whereas burglaries went down by 18% (see Table 6).

**Table 6:** The percentage difference between actual and predicted registered property offences along with effect size (Cliff’s Delta) during various phases of lockdowns in both waves in 2020 and 2021 in Chennai.

Crime/Period	Robbery	Robbery	Burglary	Burglary	Theft	Theft
	% Change	Cliff’s delta	% Change	Cliff’s delta	% Change	Cliff’s delta
*Wave One*						
CL-2020	−75.814	−1	−56.535	−1	−83.999	−1
PL-2020	−47.490	−0.944	−37.57	−0.889	−61.875	−1
Post-L-2020	7.203	0.591	17.23	0.102	17.875	0.714
*Wave Two*						
PL-One-2021	−37.095	−0.376	−3.455	−0.09	−33.987	−0.622
CL-2021	−78.358	−0.879	−44.060	−0.889	−66.331	−1
PL-Two-2021	−32.685	−0.341	−13.295	0.065	−43.678	−0.686
Post-L-2021	23.4	0.25	−18.5	−0.375	15.6	0.75

*CL* complete lockdown, *PL* partial lockdown, *Post-L* post-lockdown.

Thus, the decline in all three property offences was steeper in Chennai when compared to TN in most periods of restrictions. The relationship of the downward trends in property between the two geographical entities of TN and Chennai is closely tied to their changing mobility trends in the respective community spaces of the regions.

## Discussion

The impact of natural disasters, industrial accidents, the assassination of dignitaries, and civil unrest on mobility rarely tends to stretch to more than a few weeks. However, the various lockdown phases in waves one and two of the pandemic extended for almost two years. This inhibitory measure in the century extraordinarily modified every sphere of human activity, transcending into newer frontiers of lifestyle such as working from home, online education, webinars, and virtual meetings.

**Fig. 5:**
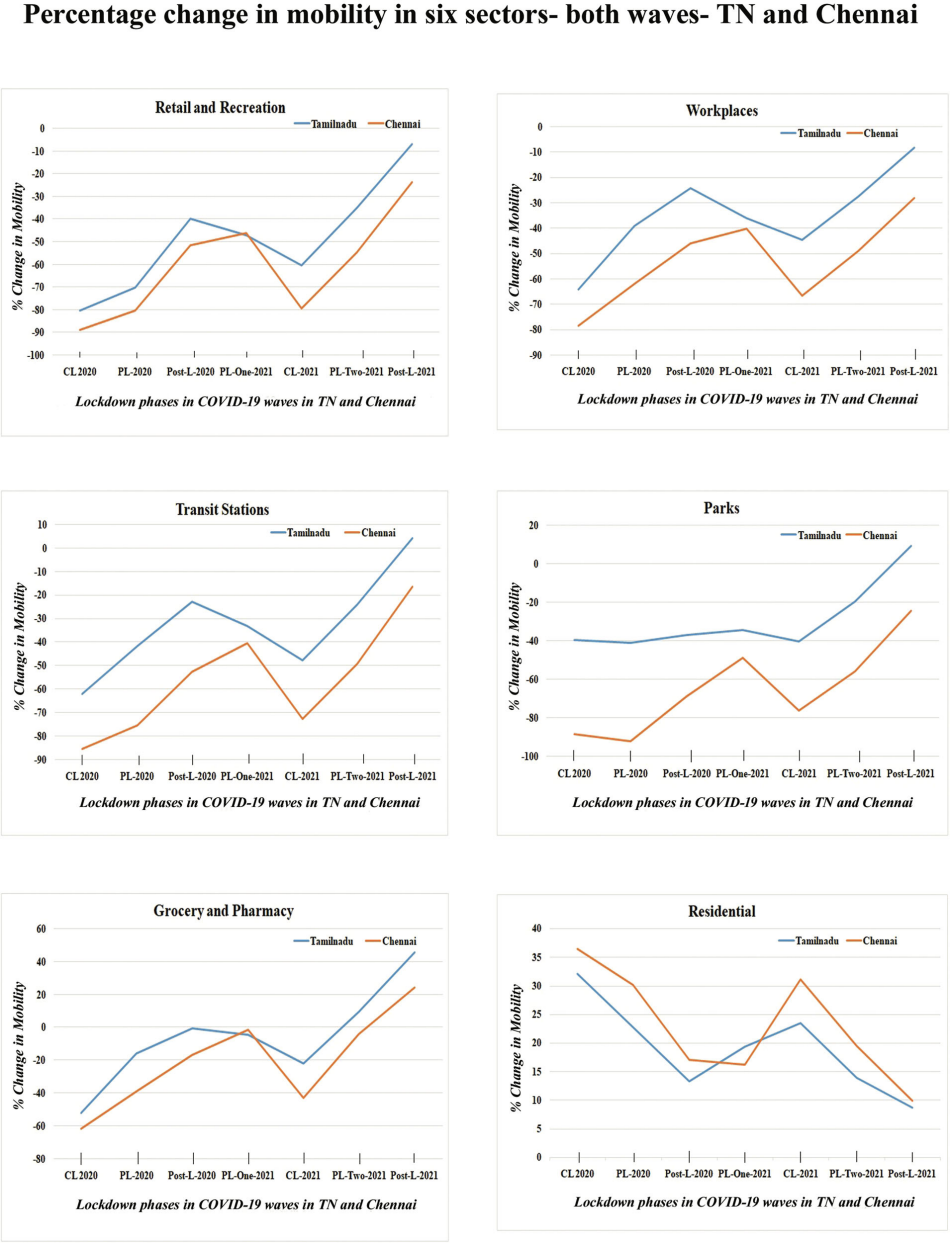
Mean percentage change in mobility from the baseline in six community spaces during various phases of lockdown in both waves in 2020 and 2021 in Tamil Nadu and Chennai City. The *X*-axis depicts the various stages of lockdown in discrete intervals (CL complete lockdown, PL partial lockdown, Post-L post-lockdown), and *Y*-axis shows the mean percentage change in mobility from baseline in different community spaces.

The most restrictive phase in the entire two-year pandemic period was CL-2020, when every community space (except residential) in TN witnessed the highest reduction in mobility. The most substantial one was in the sector of retail and recreation in TN. Similarly, Chennai, too witnessed the same trend as TN during CL-2020, with a more noticeable reduction in other spaces such as parks and transit stations. The public space of groceries and pharmacies exhibited a different trend in reduction when compared to other spaces, both in TN and Chennai (see Fig. 5); this is the only sector that witnessed increased mobility from the baseline (pre-pandemic time) when the restrictions were relaxed or completely removed during the second wave. Again, this is quite intuitive to understand as increased mobility signals higher movement of people, especially in the sector where pharmacies are located. It was indispensable as people had to step out more often to get medicines and related items during pandemic times than during regular times. Further, this public space concerns the very sustenance of life; hence, the overall mobility pattern in this sector is different from others, such as retail and recreation, workplaces, and transit stations.

**Fig. 6:**
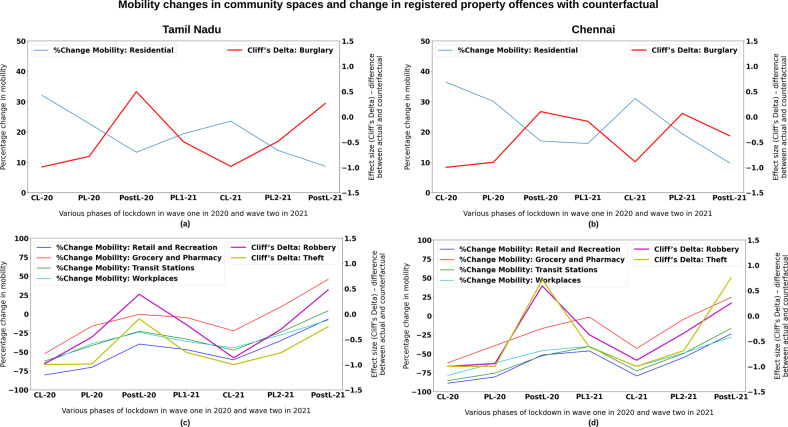
Relationship between change in mobility and its impact on registered property offences. Association between the mean percentage change in mobility from baseline in different community spaces and change in actual registered property offences such as thefts, burglaries, and robberies with the counterfactual as expressed in effect size (Cliff’s Delta) across various phases of lockdown in both pandemic waves in Tamil Nadu (Left Panel—**a** and **c**) and Chennai City (right panel—**b** and **d**). The top panels are Residential vs. Burglary offences. The bottom panels are Other community space vs. Thefts and Robberies. The *X*-axis depicts the various stages of lockdown in discrete intervals (CL complete lockdown, PL partial lockdown, Post-L post-lockdown), and *Y*-axis shows the mean percentage change in mobility from baseline in different community spaces.

In the analysis of the influence of mobility on crime, it is revealed that all three property offences have been uniformly impacted. They have witnessed a drastic reduction during CL-2020 and CL-2021. However, the magnitude of the impact on burglary was less than on robbery and theft cases, both in TN and Chennai. The percentage decline in robbery and theft in TN in CL-2020 was 83 and 80%, as against 69% for burglary; similarly, in Chennai, the corresponding percentage decrease was 75% and 84%, as against 56%. It is clearly seen that in most spatial domains, the altered mobility concomitantly shows that routine behaviour of people caused the opportunity structure to change, which corroborates the steep fall in property offences. The synchronous trends can be seen in Fig. 6. After the enforcement of social distancing norms and the drastic reduction of interaction between individuals, people living in apprehension severely curtailed opportunities for criminal offending (Stickle and Felson, 2020).

In order to comprehend the mechanics of the occurrence of robbery, theft, and burglary, it is essential to understand the nuanced differences among these offences. Though all these crimes come under the umbrella of property offences, theft cases here only refer to property lost outside the premises of a building; in such cases, there is no criminal trespass by the offender into the victim’s premises. These offences primarily include vehicle theft, chain snatching, and mobile phone snatching; in rural areas, they also include wire theft and motor and water pump theft. Robbery cases could be either outside or inside the premises, but a significant number of robberies in TN occur outside. In contrast, burglary cases are necessarily inside the premises of the building, and in this study, most cases are residential burglaries.

In the eight-time windows, the mean percentage mobility change in three sectors, namely retail and recreation, workplaces, and transit stations, is synchronous with the decrease and increase of actual registered robbery and theft cases in TN and Chennai (see Fig. 6). This, again, is in alignment with the Crime Opportunity Theory. The offenders were not motivated to commit the crime because of the meagre reward (lower number of likely victims given the reduced mobility) and increased risk (higher probability of getting caught due to a sparse crowd), specifically in these spheres of activities.

A burglary occurs inside the premises, where guardianship plays an important role. Guardianship is a critical factor in the fall and rise in burglary cases during lockdown phases. Crime trends in burglary cases are contemporaneous with mobility trends in residential areas, which directly conform to RAT. The graphs in Fig. 6 clearly exhibit the inverse trend relationship of increasing mobility with decreasing burglaries in all the lockdown phases for both TN and Chennai. Chennai witnessed an enhanced guardianship than TN and correspondingly relatively lower burglary levels. The more noticeable change observed was the temporal shift in crime occurrences (Chen et al., 2021). The closure of all activities made no distinction between weekdays and weekends. The holiday effect on crime that existed in the pre-pandemic period was absent during the pandemic period. Most of the days resembled holidays during lockdown periods and drove down offences, particularly burglaries, given increased guardianship.

Speaking of urban–rural differences, the predominantly urban city of Chennai experienced a more pronounced reduction in mobility from the baselines in all eight-time windows of differing intensities of lockdown across all five community spaces than TN, which has a larger rural landscape. There was an escalation in the percentage change in mobility from the baseline in the residential sector, as expected. The magnitude of such an increase was higher in Chennai than in TN. Several factors may have contributed to relatively reduced mobility in most community spaces and increased mobility in the residential sector in Chennai than in TN. The substantially higher per capita number of doctors, beds, hospitals, and medical shops in Chennai could perhaps be the reason for a relatively more significant drop in mobility from the baseline than the corresponding levels in TN in public spaces. (TN Government Health Department, 2019). This is because nearer and more accessible healthcare facilities imply less travel and movement for people. Chennai surpassed all other districts in TN on various pandemic counts, thus making people want to stay home to avoid infection. Despite being equipped with better health infrastructure, the city experienced a much more devastating impact, such as a surging infection rate, an excessively higher number of deaths, and higher hospital admissions than other districts (Lewnard et al., 2022). These factors, along with a higher literacy rate of 90.18% in Chennai compared with 80.09% in TN (Government of India, 2022), were complemented by better enforcement of SAH orders by police on account of higher per capita officers and police stations, which also may the reason to drive down the mobility further. The relatively higher magnitude of change in mobility in Chennai than in TN also sees correspondingly lower levels of recorded property offences in the various phases of lockdown when restrictions were in place.

A multitude of theories of crime highlight the importance of opportunity in the occurrence of crime (examples include routine activities theory, offender search theory, and environmental design theory). Such opportunities are said to occur when a set of conditions are met. For instance, in routine activity theory, the cooccurrence of a “potential offender, a suitable target, and the absence of a capable guardian” is seen as a precondition for the crime. Our study explains and empirically shows that such conditions are more probabilistically frequent with increased mobility. In this study, we adjusted for mobility. The authors use mobility as a spatio-temporal indicator of expected crime and, therefore, in the context of this study, as a covariate. This variable *mobility*, measured across various public spaces in the gamut of all phases of lockdown, directly or indirectly impacts various other criminogenic factors that are latent and not measurable. Alternatively, one can say the covariate “mobility” correlates to the factors outside the scope of this study contributing to crime occurrence and registration. The rationale for adding such a catchall nuisance variable is that it will contribute to some of the explanation of the output, namely crime registered. Its consequent impact on the relationship inferred between variables of interest in this study, and the output is what we refer to as adjusting for mobility.

The most marked impact, which is of paramount concern, is that when all the curbs were removed during the Post-L phases in both waves, the actual robbery cases shot up by noticeable percentages compared with the counterfactual both in TN and Chennai. Notably, robberies rose in TN by 56% (ES 0.47) and in Chennai by 23.4% (ES 0.25), whereas mixed trends were noticed in burglaries and thefts in TN and Chennai. While theft and burglary occur without the knowledge of victims, there is an element of force or intimidation, insinuating the desperation of the offender. This increase could perhaps be attributed to the high unemployment rate during the lockdown phases in both waves. The regular unemployment rate, which was in the range of 5–10%, saw an unprecedented increase in April, May, and June 2020 in TN—it increased to 49.8%, 33.2%, and 12.2%, respectively (Centre for Monitoring Indian Economy [CMIE], 2021). The critical study of Chiricos (1987) established a positive and significant relationship between unemployment and property offences. Similarly, the studies of Raphael and Rudolf (2001) and Öster and Agell (2007) reiterate the statistically and economically significant effect of unemployment on property offences.

## Research contributions and limitations

The primary contribution of the research is a deeper understanding of the impact of the pandemic on crime, specifically regarding the need for immediate change in the protocol and charter of duties of police and related criminal justice administration organs. When most curbs were removed during the Post-L phase in 2021, the first responders were still grappling with pandemic-related commitments as the infection and fatality rates, though dropping, were not nearing zero. During the pandemic, the changed operating procedures for law enforcement could not be reset immediately to the original levels of functioning and protocol; the changes included minimal contact with the public, limited arrests of the only severe and grievous offenders, limited court functioning, online proceedings; and the depopulation of prisons, jails, and police-station lock-ups.

Though mobility in public spaces increased in the post-lockdown phases compared to the complete and partial lockdown periods, it remained lower than in the pre-pandemic period. However, in these post-lockdown phases, the upsurge in property offences, especially robberies in this phase, required the police to make an appropriate quick re-ordering of their protocol and priorities.

The paper’s novelty also lies in introducing an ARNN model and its accuracy in prediction and robustness to handle wide-fluctuating data with multi-seasonality. With easier access and the availability of higher computational capabilities, this method is set to gain prominence among criminologists, policymakers, and social scientists as a forecasting tool for time series analysis. This ARNN model for predicting time series is user-friendly and requires very little manual intervention from the researcher in specifying the functional form. Further, it does not assume the error term to be Gaussian; instead, it assigns an appropriate continuous or discrete likelihood function depending on the dataset. Unlike the conventional methods, ARNN’s efficacy is dependable even when sparse data has missing values. It can make predictions even when there is little information about past observations as it learns from other related time series. The model is also versatile in terms of the nature of data that can be used for forecasting; for example, it can also use real-time forecasting using video-surveillance data (Shah et al., 2021). It can also be used for spatial forecasting of crime with site-specific features extracted from web mapping platforms (Kang and Kang, 2017).

There are two major limitations to this study. Under-reporting of property offences even during regular times has been discussed as one of the limitations of the data. However, during the pandemic, situational factors such as apprehension of infection shared commitment of police, and victims’ accessibility to the police might have driven down crime registration over and above the influence of mobility. While this effect would only amplify our findings, it has not been quantified.

Though wave two of the pandemic was more devastating and disastrous than the first, there was relatively higher mobility in wave two. The presumed reasons are that (i) people did not fully comply with the lockdown orders in wave two, (ii) the fear of an unknown COVID-19 in wave one was absent in wave two, and (iii) the vaccination drive and advancement in methods to treat COVID-19 patients. The present research would have been more comprehensive if qualitative studies were included in determining the actual reasons for the variations in mobility during both waves.

## Conclusion

First, the study covering a longer span of almost two years combined with the historical data of 10 years enabled the researchers to conclude a clear, direct relationship between mobility and the incidence of property offences. During the strict and stern SAH orders, a very large ES difference was noticed. Similarly, medium to large ES differences was seen during the mild SAH orders.

Second, when the curbs were removed in 2021, it was expected that the recorded levels of property offences would return to normalcy. However, after adjusting for mobility, there was a marked increase in robberies in TN and Chennai compared with the counterfactual. The changes in thefts and burglaries showed mixed trends in urban and rural places in the post-lockdown period. This could perhaps be due to the impact of the pandemic-induced lockdown on the economic status; there was an unprecedented increase in unemployment rates during the period of SAH order in wave one and a moderate increase in wave two.

Third, there was no substantial urban-rural difference in the impacts of the pandemic-induced SAH orders on property offences in TN, which has a sizeable rural population, and Chennai, which has an overwhelmingly urban population. However, Chennai, which is better equipped with the resources of first responders than TN, had better enforced the lockdown as reflected in mobility and the concomitant effect on the relative decrease in property offences as well.

Fourth, for researchers and practitioners in the field of criminal justice, ARNN would serve as one of the most accurate methods of forecasting crime, given the nature of data, availability of computing power, and ease of prediction without much manual intervention.

Finally, the research findings highlight the need for police departments to gear up preventive crime work as escalated levels of robbery in TN and Chennai were quite marked when the restrictions were removed, especially by quickly resetting their protocol and charter of duties which changed considerably during the pandemic time.

## Supplementary information

Code

## Data Availability

The data that support the study’s findings are available from the State Crime Records Bureau of the Tamil Nadu Police Department. The author does not have permission to share the data for public use. However, upon a reasonable request, the data can be made available for research purposes after the researchers get consent from the Tamil Nadu State Crime Records Bureau. The data are available from Harvard Dataverse 10.7910/DVN/PWFE3H.
